# The role of periradicular infiltration in dorsal root ganglion stimulation for chronic neuropathic pain

**DOI:** 10.1007/s00701-021-04745-y

**Published:** 2021-02-09

**Authors:** H. Sievert, G. S. Piedade, P. McPhillips, J. Vesper, P. J. Slotty

**Affiliations:** 1 Medical School of the Heinrich-Heine-Universität Düsseldorf, Düsseldorf, Germany; 2 Department of Neurosurgery, Heinrich-Heine-Universität Düsseldorf, Düsseldorf, Germany; 3 Department of Functional Neurosurgery and Stereotaxy, Heinrich-Heine-Universität Düsseldorf, Düsseldorf, Germany

**Keywords:** Periradicular infiltration, Dorsal root ganglion Stimulation, Neuromodulation, Chronic pain

## Abstract

**Background:**

Targeting the correct spinal level is essential in dorsal root ganglion (DRG) stimulation. Anatomical selection of the DRG alone is not ideal since the pain area is not necessarily confined to the borders of the dermatomes. This study aims to establish the role of periradicular infiltration therapy (PRT) in the preoperative assessment of the correct level for DRG stimulation performed under general anesthesia.

**Method:**

We report a prospective study of 20 patients selected for DRG stimulation and submitted to a PRT for identification of the spinal level. Lead implantation for the stimulation trial occurred under general anesthesia: 19 patients experienced positive results and underwent implantation of the pulse generator. All patients suffered from chronic neuropathic pain unresponsive to best medical treatment. PRT levels were compared with the levels targeted with DRG leads. Patients were followed for up to 12 months; pain intensity and coverage of the painful area were assessed.

**Results:**

In 12 patients, the trial leads were placed on the same level as previously tested positive by PRT. In 6 patients, leads were placed in the PRT target and additionally in adjacent spinal levels. In one case, the selected target for the trial diverged from the PRT target because of intense fibrosis in the chosen level. Coverage of the target area of at least 50% was achieved by two-thirds of the patients. For the six subjects with additional implanted leads as a consequence of the PRT results, 80% achieved a coverage of at least 50%. A total of 47.4% of the patients achieved sustained significant pain relief in the last follow-up. None of the patients needed a repeated surgery for implantation of additional leads.

**Conclusions:**

PRT is a helpful tool to confirm the stimulation targets. A PRT preceding the stimulation trial is an additional opportunity to optimize the coverage of the target area with stimulation-induced paresthesia for patients operated under general anesthesia.

## Introduction

The therapy of chronic neuropathic pain remains a challenge today. To date, only 30 to 40% of patients with neuropathic pain can be treated satisfactorily with medication alone [3]. Conventional spinal cord stimulation (SCS) has been used successfully since 1967 to treat neuropathic pain. Yet, the results are not completely satisfying in all patient populations. The dorsal root ganglion (DRG) offers a relatively new target for neuromodulation due to its important role in the development and maintenance of chronic pain, as well as its anatomically convenient accessibility. DRG stimulation represents an effective supplement to SCS by providing precise, targeted stimulation even of discrete pain regions in areas that are difficult to reach with conventional SCS and improved patient outcomes for certain pain disorders [5]. The ACCURATE study has shown that DRG stimulation provides long-term, sustained pain relief for specific pain disorders and painful regions, being superior to conventional tonic SCS in 3 and 12-month studies [2]. Targeting the correct spinal level is essential for a successful pain treatment. Moreover, the number of electrodes is limited to 4 by the contacts of the implantable pulse generator and, each additional electrode increases the risk of surgical complications, such as infection or dislocation.

The initial selection of the correct DRG for stimulation is mostly based on the pain distribution among dermatomes. After a spinal level is targeted, a DRG stimulation lead is normally implanted with an extension lead externalized for a stimulation trial. If the patient benefits from this trial, the implantable pulse generator (IPG) can be inserted in a second procedure. Alternatively, both leads and IPG can be implanted in the same procedure, all-in-one. The issue is that an anatomical selection of the DRG alone is not ideal since the pain area is not necessarily confined to the borders of the dermatomes. Additionally, dermatomes often show unique distributions with overlap.

In the literature, selective radiofrequency (RF) stimulation of the DRG has been discussed as a method for predicting the correct spinal level for stimulation, possibly giving important information for lead implantation in a stimulation trial or even in an all-in-one procedure [4]. Only two case studies on RF stimulation prior to DRG stimulation have been published so far; no standard preoperative procedure for DRG stimulation has been established yet. As a result, most surgeons have their own approach to solve the problem of pre-surgical targeting. A frequently used alternative is a CT-guided periradicular infiltration therapy (PRT). This procedure uses local anesthetics and can be easily performed on the preoperative day, efficiently helping the surgeon to choose the spinal level for DRG stimulation. There are no valid data associating PRT results with DRG outcomes so far. This study aims to establish the role of PRT in a preoperative assessment of the correct level for DRG stimulation regarding the coverage of the painful area with stimulation-induced paresthesia.

## Methods

This is a prospective single-arm study that evaluates the outcomes of patients undergoing implantation of a DRG stimulation system. Twenty patients scheduled for DRG stimulation were prospectively observed between 2016 and 2018. All patients were at least 18 years old with an indication for DRG stimulation due to a chronic pain disorder refractory to best pharmacological treatment. No patients were excluded due to previously known intolerances to local anesthetics administered as part of PRT or to other contraindications to the procedure.

The baseline pain assessment was performed using a visual analog scale (VAS). On the same day or in the following days a PRT of the presumptive affected DRG was performed [6], the target level was chosen on a clinical basis and in some cases multiple levels were chosen because the clinical examination by the responsible surgeon showed that the pain extended over several dermatomes. In our institution, a diagnostic PRT consists of the injection of bupivacaine 2.5 mg/mL. In cervical roots, 2 mL is the maximal injected volume, while in lumbar roots generally 3 mL is used. Dexamethasone is injected with bupivacaine only in therapeutic PRTs and was therefore not used. Bupivacaine has an elimination half-life of 143 min following epidural administration [1] and the PRT is performed at least 24 h before lead implantation. Patients were clinically evaluated by the responsible surgeon up to 2 h after the PRT, sensibility to light touch was assessed with either a tissue or cotton. During the consultation, the patient was asked about pain relief and to what extent the painful area was covered by the PRT (completely, partially, not at all). PRT testing was considered positive if patients responded with pain relief in the corresponding painful area. Complete pain relief was not required for a positive PRT assessment, as the goal was to find the appropriate level of stimulation and not to achieve complete pain relief with PRT. If the anesthetized region and the pain region were not congruent, another PRT of a different, usually adjacent spinal level was performed usually 1 day after the first one at the discretion of the responsible surgeon. After congruent PRT results to the painful area, lead placement was performed for trial stimulation or exceptionally in an all-in-one procedure. At the discretion of the surgeon, additional leads were implanted in adjacent levels if there was insufficient coverage of the pain region with the PRT effect. Negative PRT results were not considered exclusion criteria for a DRG trial.

For the trial period, one to three leads were placed using a minimally invasive epidural approach under general anesthesia. No intraoperative paresthesia testing was done. Leads were anchored to the muscular fascia and were attached to an external trial stimulator using externalized extensions; stimulation was provided for 3 to 7 days. At the end of the trial, a new evaluation of the pain condition was performed using VAS. With a pain reduction of 50% and/or objective functional improvement of the patient, the trial was considered successful and the implantation of the IPG was performed (Proclaim DRG; Abbott Neurological, St. Jude Medical, Minneapolis, MN, USA). Patients with an increased surgical risk as well as patients with a clearly positive PRT result according to the experience of the responsible surgeon underwent all-in-one surgery. After the implantation of the complete neurostimulation system, the patient was interviewed in the regular out-patient visits within 1 week, as well as 1, 3, 6, and 12 months postoperatively using VAS, questionnaires, and pain/paresthesia maps. The existence of paraesthesia in the previously painful area as well as the percentage of painful area covered with paraesthesia was documented. Patients with a pain relief of at least 50% under DRG stimulation were considered responsive.

All study elements were approved by the local ethics committee, and each patient gave written informed consent prior to the beginning of any study activities.

## Results

**Fig. 1:**
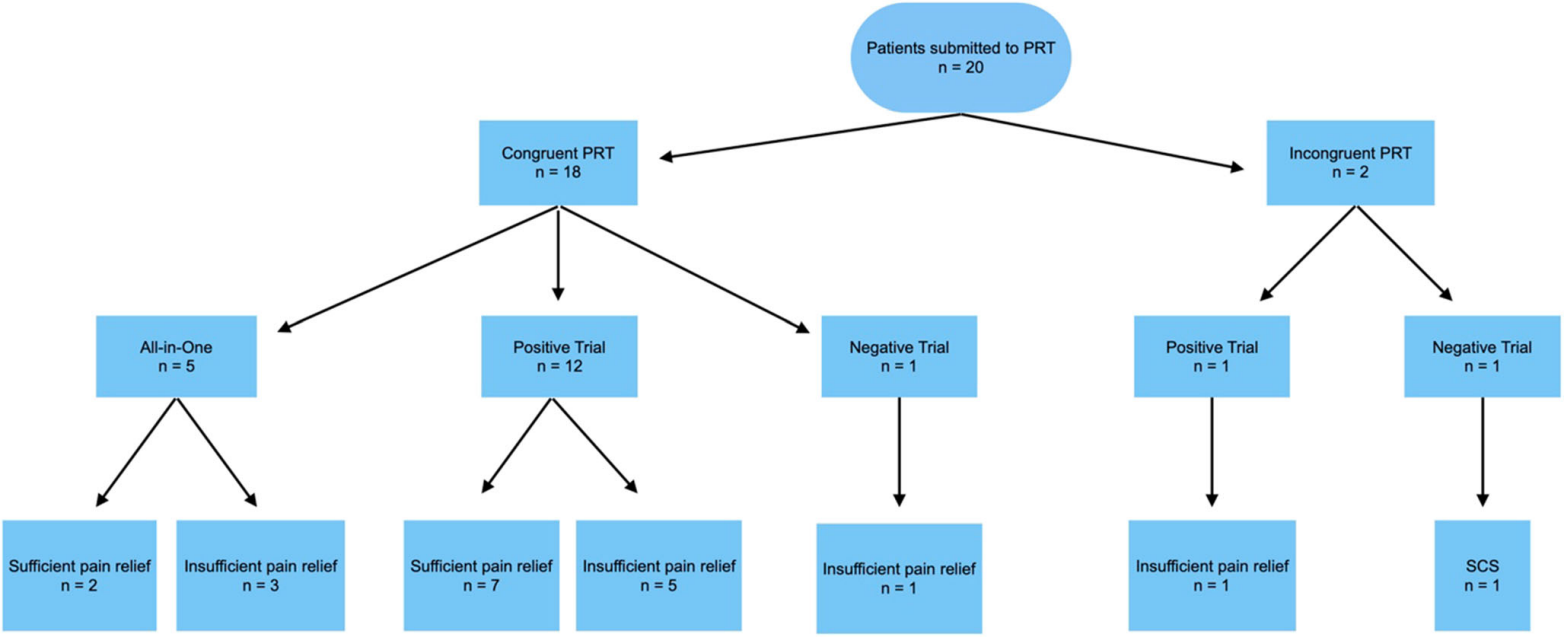
Flow diagram of included subjects

Twenty patients with the indication for DRG stimulation were evaluated regarding pain development (Fig. 1). Preoperative PRT was performed in all patients; no complications were observed. When results were not clear or incongruent with the painful area, a second PRT was performed, and it was the case of 4 patients; for a congruent result, at least one PRT should be congruent. Overall, five patients were affected by CRPS, four patients by FBSS, and most patients had another form of postsurgical neuropathic pain. Mean age was 54.8 years in the group; mean follow-up time was 10.9 months.

From overall 20 included patients, PRT was congruent with the pain region in 18 cases (90%). The two patients with incongruent results were however trialed for DRG because the pain region was clearly related to a very specific dermatome—one case did not achieve relevant pain relief during the trial and was later treated with an SCS; the other one was responsive during the trial and progressed to IPG implantation (patient 14). Because this single patient underwent an SCS, DRG stimulation was performed on 19 patients during this study.

In the 18 patients with congruent PRT, five patients were selected for an all-in-one implantation at the discretion of the treating surgeon because the PRT yielded an adequate coverage of the painful area with a significant pain reduction. Out of this group, only two patients (40%) reported relevant sustained pain relief under DRG stimulation.

The remaining 13 patients with congruent PRT that were not considered for an all-in-one implantation were submitted to a trial; 12 patients had a positive trial. These subjects had a sustained significant pain relief under DRG stimulation in 53.8% of the cases in the last follow-up (7/13). The only congruent PRT result but insufficient trial result was selected for the implantation of the IPG at discretion of the treating neurosurgeon for reasons that include significant functional improvement. No significant pain relief was achieved in this particular case. Considering now all patient groups, mean reduction in pain intensity under DRG stimulation was 31.7%; a total of 47.4% of the patients achieved sustained significant pain relief in the last follow-up (9/19).

**Fig. 2:**
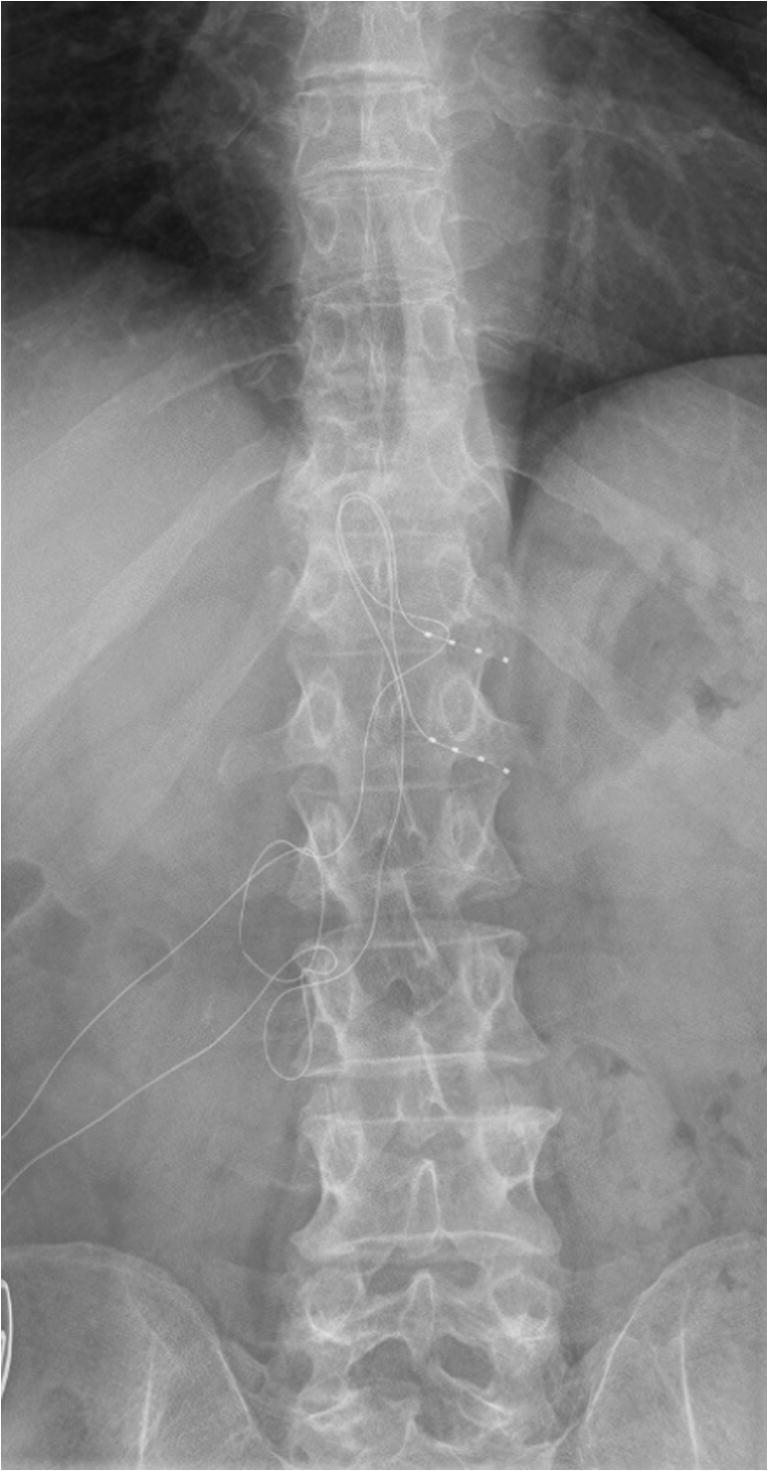
DRG leads implanted in Th12 and L1 on the left side, the patient suffered from chronic pain after a salpingectomy

In 11 patients, the trial leads were placed on the same level as previously tested positive by PRT (Table 1). In 6 patients, leads were placed in the PRT target and additionally in adjacent spinal levels, meaning that the PRT modified the original plan. In 15 patients, the leads were implanted on the same level as previously tested in the trial; in 2 patients, additional leads were implanted as a consequence of the trial results (patients 8 and 10) (Fig. 2). In the particular case of patient 13, the implantation of a DRG lead in S1 was technically not possible because of fibrosis, and the patient had a lead in L5 implanted.

**Table 1:** PRT and DRG levels, clinical outcomes

	Sex	Age	Diagnosis	PRT level	PRT congruence	DRG level	VAS Baseline	VAS 1 mo	VAS 3 mo	VAS 6 mo	VAS 12 mo	Coverage
1	M	58	FBSS	S1 left	+	S1 left	9	5	10	10	9 (0%)	30%
2	F	41	Pain after peripheral nerve injury	C6, C7 right	+	C6, C7 right	10	-	7	5 (−50%)		100%
3	F	27	FBSS	S1 left	+	S1 left	8	-	0	0	0 (−100%)	100%
4	F	52	CRPS I	C6 right	+	C6 right	7	5	7	6	7 (0%)	90%
5	F	29	CRPS II	L5 left	+	L5, S1 left	6	1	-	2	3 (−50%)	20%
6	M	56	Postarthroplasty	L3, L4 right	+	L2, L3, L4 right	5	5	7	4	2 (−60%)	100%
7	M	64	FBSS	L2 right	+	L2 right	7	7	7	1	3 (−57%)	-
8	M	44	CRPS II	L4 left	+	L4, L5, S1 left	10	-	9	10	9 (−10%)	-
9	M	42	CRPS II	L5 right	+	L5, S1 right	8	6	-	8	8 (0%)	50%
10	F	35	Postthoracotomy	Th11 right	+	Th9, Th10, Th11 right	8	5	5	3	2 (−75%)	100%
11	F	77	Postarthroplasty	L3, L4 right	+	L3, L4 right	8	9	8	4	3 (−63%)	-
12	M	68	Postarthroplasty	L3 right	+	L3, L4 right	3	2	6	-	6 (+50%)	0%
13	M	45	FBSS	S1 left	+	L5 left	9	-	-	-	7 (−22%)	-
14	M	80	Postarthroplasty	L3, L4 right	−	L3, L4 right	8	5	6	7	8 (0%)	-
15	F	51	Postsalpingectomy	Th12, L1 left	+	Th12, L1 left	9	7	9	-	9 (0%)	0%
16	M	69	Postarthroplasty	L3, L4 right	+	L3, L4 right	7	2	2	3	6 (−14%)	100%
17	M	82	Postarthroplasty	L2, L3 left	+	L2, L3 left	8	6	4	4	7 (−13%)	100%
18	F	49	Posttraumatic	S2 both	+	S2 both	9	0	1 (−89%)	-	-	-
19	M	79	Postherniotomy	L1, L2 right	+	L1, L2 right	10	0	0	5 (−50%)	-	-
20	F	49	CRPS I	Th9, Th12 right	−	None	8	-	-	-	-	-

A second PRT was done in patients 1 (S1 left), 2 (C6 right), 10 (Th11 right), and 17 (L2 and L3 left). All-in-one procedures were the case of patients 5, 11, 12, 16, and 17. Implantation of a DRG lead in S1 was technically not possible in patient 13 due to fibrosis. *PRT* periradicular therapy, *DRG* dorsal root ganglion, *VAS* visual analog scale, *FBSS* failed back surgery syndrome, *CRPS* complex regional pain syndrome

Data to coverage of the painful area with paraesthesia was available for 12 patients, all of them with a previous congruent PRT result. Two-thirds of them reported a coverage of the target area of at least 50%. For the six patients with additional implanted leads as a consequence of the PRT results (patients 2, 5, 6, 8, 9, and 10), 80% achieved a coverage of at least 50%, with data being unavailable for patient 8.

A total of 7 patients underwent revision surgery, which included broken leads and lead defects, among other causes. One patient died before the end of the study unrelated to the DRG system or surgery, the remaining 18 implanted patients were observed over a period of 12 months.

## Discussion

The aim of the study was to investigate whether the preoperative periradicular therapy is eligible in a preoperative protocol for identifying the correct spinal level for DRG stimulation regarding the coverage of the painful area with stimulation-induced paresthesia. Compared to the past case studies on methods for predicting targets for DRG stimulation by Zuidema et al. [7] on retrograde transforaminal paresthesia mapping, with 3 patients with groin pain, and by Hunter et al. on radiofrequency stimulation, with 4 patients with postamputation pain of the lower extremity [4], a considerably larger number of patients could be examined. Similarly, the selection of patients investigated in this study was not limited to an underlying disease or localization of pain. The study thus provides a good representative picture of the patient population of neuropathic pain. In comparison to the mentioned studies, the present study enabled an analysis of a longer-term stimulation result after successful PRT testing.

In our department, PRT has become the standard of care in almost all patients being screened for DRG for confirmation of the target level. Bupivacaine is usually preferred and has a longer elimination half-life than lidocaine. Lead implantation occurs on the following day so that no analgesic effect of the PRT should be present. In this study, however, all lead implantations were done under general anesthesia without paresthesia control. Even when the initial PRT does not cover the entire painful area, it orientates the surgeon when choosing the target DRG. If the first PRT result is not congruent with the painful area, a second PRT may be helpful, which was the case of three patients in this study. In case of insufficient coverage after PRT, the direct implantation of another leads in the trial without prior testing becomes more justifiable when a first PRT confirmed at least partial improvement. As a single-arm study, no comparisons can be made with the coverage rates of a control group that did not undergo a preoperative PRT. Our study was, however, able to show that the PRT results modified the original targets established by the responsible surgeons based on anatomical landmarks in a considerable number of patients. It is true that insufficient coverage can also be detected in the trial phase, but the preoperative PRT turns the trial into a second opportunity to evaluate the adequate coverage of the painful area before implantation of the definitive system. Unfortunately, we did not find any references regarding the incidence of second or even third procedures for the implantation of new DRG leads after the implantation of the IPG because of insufficient pain coverage. It is intuitive, however, that a preoperative PRT could reduce the length of hospital stay and the risks of new surgical procedures because more affected levels are earlier identified additionally to the clinically inferred ones. It might offer an additional option to reconsider the neuromodulation strategy for every individual patient. In this study with 19 subjects submitted to DRG stimulation, a second operation for implantation of new leads did not occur.

Not as intuitive is the possible predictive value of preoperative PRT over the outcomes of DRG stimulation. These therapies have different mechanisms of action, but such a relationship would be of considerable interest, as it might indicate which patients would not benefit from DRG stimulation—whose technique for lead placement is particularly more difficult when compared with traditional spinal cord stimulation. For a matter of comparison, the positive predictive value of a successful trial for sustained significant pain relief achieved 53.8% in this study. As only one patient had a negative trial and was submitted to DRG stimulation later, nothing can be said about its negative predictive value based on these data.

Particularly interesting is the case of the five patients submitted to an all-in-one implantation of DRG leads following a very successful PRT testing. In these cases, when PRT results were most promising considering adequate coverage and reduction of pain intensity, the positive predictive value for final significant pain relief was only 40% and 50% for coverage of at least 50% of the painful area. This result regarding pain reduction is lower than the predictive value of a trial (53.8%), which remains as the gold standard for the selection of patients for implantation of the definitive system. The predictive value regarding coverage of the painful area was also lower than the value obtained considering all 12 patients with available coverage data (67%). The indication for an all-in-one implantation of DRG leads is given at discretion of the responsible surgeon and should be specially considered in patients with higher surgical risk, but data of this study with a limited sample size supports a stepwise approach with a stimulation trial—independent of how promising PRT results are.

### Limitations

This study evaluated only the congruence of PRT effect with the painful area and not the effect of PRT over the pain intensity. No conclusions can be drawn regarding its predictive value to stimulation outcomes. It is however relevant to mention that the variability of PRT results is influenced by physician experience and technical aspects, such as anesthetics used and addition of steroids. Therefore, insufficient pain relief after PRT would not change our indication for a DRG trial, as it was the case with patient 14. The inclusion of PRT in our clinical routine is independent of its positive predictive value over final clinical outcomes.

## Conclusion

The success of the DRG stimulation depends on the correct lead placement, and PRT is a helpful tool to confirm the stimulation targets. A PRT preceding the stimulation trial represents an additional opportunity to optimize the coverage of the target area with stimulation-induced paresthesia for patients operated under general anesthesia.
